# Effect of disease severity on the structure and diversity of the phyllosphere microbial community in tobacco

**DOI:** 10.3389/fmicb.2022.1081576

**Published:** 2023-01-04

**Authors:** Meili Sun, Caihua Shi, Yang Huang, Hancheng Wang, Jianjun Li, Liuti Cai, Fei Luo, Ligang Xiang, Feng Wang

**Affiliations:** ^1^MARA Key Laboratory of Sustainable Crop Production in the Middle Reaches of the Yangtze River, College of Agriculture, Yangtze University, Jingzhou, China; ^2^Guizhou Provincial Academician Workstation of Microbiology and Health, Guizhou Academy of Tobacco Science, Guiyang, China; ^3^School of Food Science and Technology & School of Chemical Engineering, Hubei University of Arts and Science, Xiangyang, Hubei, China; ^4^China Tobacco Sichuan Industrial Corporation Technical Centre, Chengdu, China; ^5^College of Tropical Crops, Hainan University, Haikou Hainan, China; ^6^College of Life Science, Yangtze University, Jingzhou, China

**Keywords:** tobacco target spot, high-throughput sequencing, microbial composition, phyllosphere, disease severity

## Abstract

Tobacco target spot is a serious fungal disease and it is important to study the similarities and differences between fungal and bacterial community under different disease severities to provide guidance for the biological control of tobacco target spot. In this study, tobacco leaves at disease severity level of 1, 5, 7 and 9 (S1, S5, S7, and S9) were collected, both healthy and diseased leaf tissues for each level were sampled. The community structure and diversity of fungi and bacteria in tobacco leaves with different disease severities were compared using high-throughput sequencing technology. The results indicated that there was a significant differences in the community structure of fungi and bacteria for both healthy and diseased samples depending on the disease severity. In both healthy and diseased tobacco leaves for all four different disease severities, the most dominant fungal phylum was Basidiomycota with a high prevalence of genus *Thanatephorus*. The relative abundance of *Thanatephorus* was most found at S9 diseased samples. Proteobacteria represent the most prominent bacterial phylum, with *Pseudomonas* as predominant genus, followed by *Pantoea*. The relative abundance of *Pseudomonas* was most found at S7 healthy samples. In fungal community, the Alpha-diversity of healthy samples was higher than that of diseased samples. In contrast, in bacterial community, the Alpha-diversity of healthy samples was lower than that of diseased samples. LEfSe analysis showed that the most enrich fungal biomarker was *Thanatephorus cucumeris* in diseased samples. *Clostridium disporicum* and *Ralstonia pickettii* were the most enrich bacterial biomarker in healthy samples. FUNGuild analysis showed that the pathotroph mode was the most abundant trophic modes. The relative abundance of pathotroph mode in diseased samples changes insignificantly, but a peak at S5 was observed for healthy samples. PICRUSt analysis showed that most bacterial gene sequences seem to be independent of the disease severity. The results of this study provide scientific references for future studies on tobacco phyllosphere microecology aiming at prevention and control of tobacco target spot.

## Introduction

Tobacco (*Nicotiana tabacum* L.) is one of the most important economic crops in China (Wang et al., 2016). However, the tobacco production is constrained by many tobacco leaf spot diseases (Zhu, 2002). In between, tobacco target spot is one of the most destructive types (Shew and Main, 1990). The pathogen of tobacco target spot is *Rhizoctonia solani*, and its teleomorph is *Thanatephorus cucumeris* (Elliott et al., 2008). Typical symptoms of tobacco target spots firstly appear on the old leaves as round watery spots, the tobacco leaves are chlorotic, and with yellow halo. Then the spots become a diammer of 2 to 20 cm, with concentric ring lines and dead spots (Sun et al., 2022). In last 5 years, tobacco target spot was the most severe and most common disease happened in tobacco in China with losses reaching up to 100% (Yang et al., 2014). It believes that a better understanding of pathogens from a plant microbial community perspective may lead to other ways of controlling disease in a more sustainable manner.

Changes of phyllospheric fungi and bacteria can potentially be used to evaluate the effects of efficient disease management (Huang et al., 2021a). With the development of sequencing technology, the number of studies on phyllosphere microorganisms has gradually increased in recent years, creating a new era of phyllosphere microbial community research (Hassani et al., 2018; Fitzpatrick et al., 2020; Gong and Xin, 2020). Plant phyllospheric fungi and bacteria exist in certain or all life stages of the host plant. They live in the leaves, stems, flowers, and fruits of the plant, and establish a coevolutionary relationship with the plant (Xie et al., 2021). Phylloshperic fungi and bacteria can colonize the surface (i.e., epiphytic microbiota) or interior of leaf tissue (i.e., endophytic microbiota; Agler et al., 2016). Phylloplane microorganisms include many potentially beneficial, pathogenic, and antagonistic microbes affecting plant health and productivity (Lindow and Brandl, 2003). Carlstrom et al. reported that substantial interactions exist among strains, and that a preestablished community is largely resistant to subsequent perturbations (Carlstrom et al., 2019). In tobacco, these sequencing technology have been used widely to study the structure and diversity of phyllosphere microbial community for many diseases, including tobacco brown spot caused by *Alternara altanata* (Dai et al., 2022), leaf spot caused bay *Didymella segeticola* (Huang et al., 2021a), tobacco pole rot caused by *Rhizopus oryzae* (Chen et al., 2020), and tobacco powder mildew caused by *Golovinomyces cichoracearum* DC (Huang et al., 2021b).

An increasing body of evidence suggests that the microbial communities can positively or negatively affect the fitness and health of plants by promoting disease (Bertelsen et al., 2001). The disease severity is an important factor affecting the quality of roasted tobacco. The more severe the disease is, the lower is the quality of roasted tobacco (Chen et al., 2020). Disease severity largely affects the structure and diversity of phyllosphere microbial community. The results of a previous study on the influence of the disease severity on the community structure and diversity of the tobacco leaf spot disease caused by *Didymella segeticola* showed that the community diversity of phyllosphere fungi decreased with the increase of the disease severity (Huang et al., 2021a). Similarly, Luo et al. (2017) studied the structure and diversity of phyllosphere bacterial community of pumpkin powdery mildew under different disease severities and identified that there was a significant difference in the bacterial community structure under different disease severities. Zhang et al. studied the structure and diversity of phyllosphere fungal community of pumpkin powdery mildew at different disease severities and concluded that the abundance of dominant genera in the community increased with the increase of the disease severity (Zhang et al., 2018). Based on the results reported in Zhang et al. (2019), most genera abundance decreased with the powdery mildew pathogen *Erysiphe* increasing. Although many studies on the effect of disease severity on the phyllosphere microbial community have been carried out, the effect of disease severity on the structure and diversity of tobacco target spot phyllosphere microbial community is still poor understood.

Due to serious damage of tobacco target spot, it is important to understand how the microbiota changes on tobacco leaves at different tobacco target spot disease severities. Therefore, the goals of this study were to identify the diversity and community structure of phyllosphere fungi and bacteria in tobacco target spot under different disease severities and analyzed differences depending on the disease severity. The results of this study will provide some potential guidance for the efficient prevention and control of tobacco target spot.

## Materials and methods

### Sampling sites and sampling strategy

**Figure 1:**
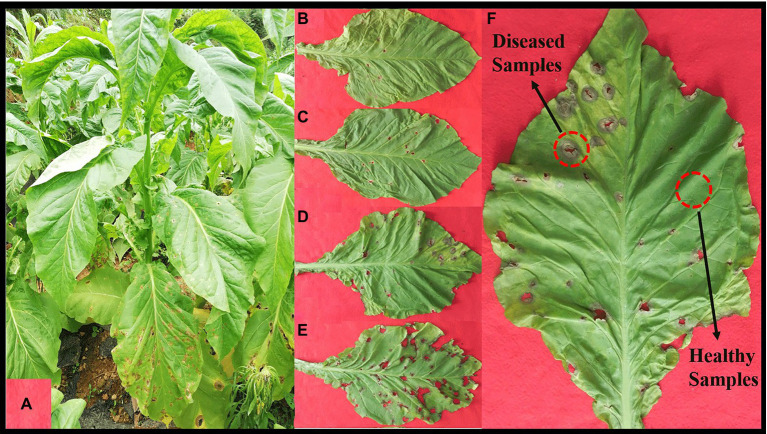
Whole plant **(A)** and leaves displaying disease severity at **(B)** S1, **(C)** S5, **(D)** S7, and **(E)** S9, respectively. **(F)**, sampling parts display of diseased and healthy samples on tobacco leaves.

In August 2021, one tobacco field with serious tobacco target spot in Zheng’an County (28°55′N, 107°44′E), Zunyi City, Guizhou Province of China, was selected as the sampling site. The 1, 5, 7 and 9 disease severities leaves were randomly selected using criteria based on the Chinese National Standard (GB/T 23222-2008). For 1, 5, 7 and 9 disease severities leaves (hereinafter referred to as S1, S5, S7, S9), the proportion of the lesion area of every leaf ranges from 0 to 1%, 11 to 20%, 21 to 40%, and 41 to 100%, respectively (Figures 1A–E). Randomly selected leaves were divided into two parts with and without obvious leaf spots and two parts (as shown in Figure 1F) were cut with sterilized scissors and put in to 50-mL sterile centrifuge tubes, which were labelled diseased samples and healthy samples. For coding samples, the letter D was used for diseased samples and H for healthy samples, which was followed by the number 1 for leaves at 1 disease severity level, 5 for leaves at 5 disease severity, 7 for leaves at 7 disease severity, 9 for leaves at 9 disease severity. For example, D1 was diseased samples from leaves at 1 disease severity. Three biological repeats were conducted. Diseased and healthy samples of the same disease severity were taken from the same leaves. Sample information was provided in Table 1. After sampling, the samples were placed into a low-temperature storage box and immediately transported to the laboratory and stored at −80°C until further use.

**Table 1:** Sample information for both diseased and healthy tobacco leaves infected tobacco target spot at different disease severity.

Disease severity	Proportion of lesion area every leaf	Diseased groups (sample)	Healthy groups (sample)
S1	<1%	D1 (D11, D12, D13)	H1 (H11, H12, H13)
S5	11–20%	D5 (D51, D52, D53)	H5 (H51, H52, H53)
S7	21–40%	D7 (D71, D72, D73)	H7 (H71, H72, H73)
S9	≥41%	D9 (D91, D92, D93)	H9 (H91, H92, H93)

### DNA extraction, PCR amplification, and high-throughput sequencing

The total DNA of each sample (*n* = 24) was extracted from the leaf tissues using the MP FastDNA SPIN Kit for Soil (MP Biomedicals, Santa Ana, United States). The purity and concentration of sample DNA were detected with gel electrophoresis. The extracted DNA was placed in a centrifuge tube and diluted to a concentration of 1 ng/μL with sterile water, which was used as DNA template. The fungal ITS1-5F region was amplified using the primers ITS1F (5’-CTTGGTCATTTAGAGGAAGTAA-3′) and ITS2R (5’-GCTGCGTTCTTCATCGATGC-3′) (Xiang et al., 2020a). Furthermore, 338F (5’-ACTCCTACGGGAGGCAGCAG-3′) and 806R (5’-GGACTACHVGGGTWTCTAAT-3′) (Xiang et al., 2020b) were used to amplify the bacterial 16S V3–V4 region. The PCR amplification was carried out based on methods described in the references (Chen et al., 2020). The PCR products were recovered by using the AxiPrepDNA gel recovery kit (AXYGEN Company, Silicon Valley, United States), eluted with Tris HCl, and detected by using 2% agarose gel electrophoresis. The amplification products were amplified using an Illumina Miseq platform and the Miseq library was constructed and sequenced on the Miseq computer.

### Data processing

After the Illumina Miseq platform sequencing, 2.3 GB raw reads were generated. All sequences were clustered according to 97% similarity with UPARSE (Version 7.1) and chimeras were filtered during operational taxonomic unit (OTU) clustering using the cluster otus command. Subsequently, unidentified bases and sequences annotated as plastids and mitochondria were removed. The OTUs were annotated with SILVA 132 and UNITE version 7.2 for the bacterial 16S rRNA and fungal ITS, respectively, set the confidence threshold to 70% (Edgar and Robert, 2013; Urmas et al., 2013). The Alpha-diversity and Beta-diversity were calculated with Qiime software (version 1.9.1) and the R statistics package (R Core Team, 2017) was utilized to draw the Principal Co-ordinate Analysis (PCoA) diagram and create the rarefaction curve (Version 2.15.3) and Venn diagrams (Version 3.0.3). The linear discriminant analysis (LDA) effect size (LEfSe) method^1^ was used to detect the potential biomarkers. The LDA = 4 discriminant score was used as the threshold value and a significant α of 0.05 was used to divide the specific biomarkers of each disease severity sample (Segata et al., 2011). Fungal nutritional modes were analyzed with the FUNGuild database (Nguyen et al., 2016). Bacterial metabolic functions were analyzed with the bioinformatics software package PICRUSt (Langille et al., 2013). Software of Cytoscape 3.9.1^2^ was used to evaluate the interactions among microbial community rate. The correlation was highly significant (*p* < 0.05) and with Spearman’s rho >0.6 and <−0.6 were considered to be correlated and selected for network analysis. (Williams et al., 2014; Meng et al., 2017).

### Statistical analysis

IBM SPSS Statistics 23 (IBM Corp., New York, United States) software was used to perform one-way ANOVA on measured data. The mean value and standard error were used to express the measured results. The Duncan’s new multiple range test was employed to test the significance of differences between different treatments. A *p* ≤ 0.05 was considered to be statistically significant.

## Results

### Quality of total fungal and bacterial sequence data

**Figure 2:**
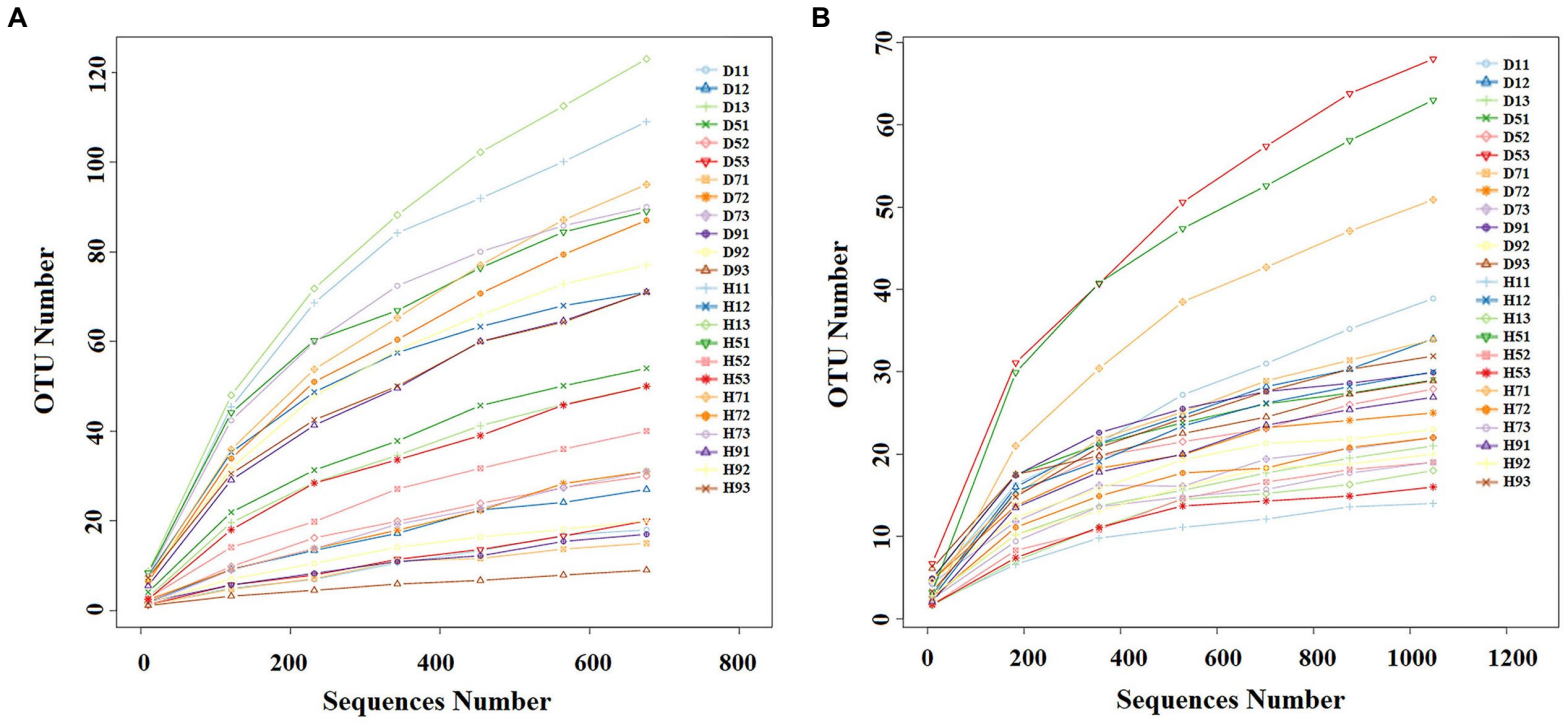
Rarefaction curves of fungal **(A)** and bacterial **(B)** OTUs across different tobacco leaf samples.

With the increase in number of sequences, the rarefaction curve gradually approached the plateau phase. When the number of fungal and bacterial sequences reaches 600 and 1,000, respectively, the rarefaction curve was saturated (Figure 2), indicating that the sequencing results reflect the structure of the phyllosphere community in tobacco leaves. A total of 2,139,800 fungal sequences were obtained from 24 samples. These sequences were classified into 484 OTUs at a 97% similarity level. For bacteria, a total of 2,184,600 sequences were classified into 271 OTUs across the 24 samples. The fungal and bacterial sequences of each sample were deposited in the SRA database under accession numbers PRJNA4081 and PRJNA4091, respectively.

### Distribution and diversity of fungal and bacterial operational taxonomic units

**Figure 3:**
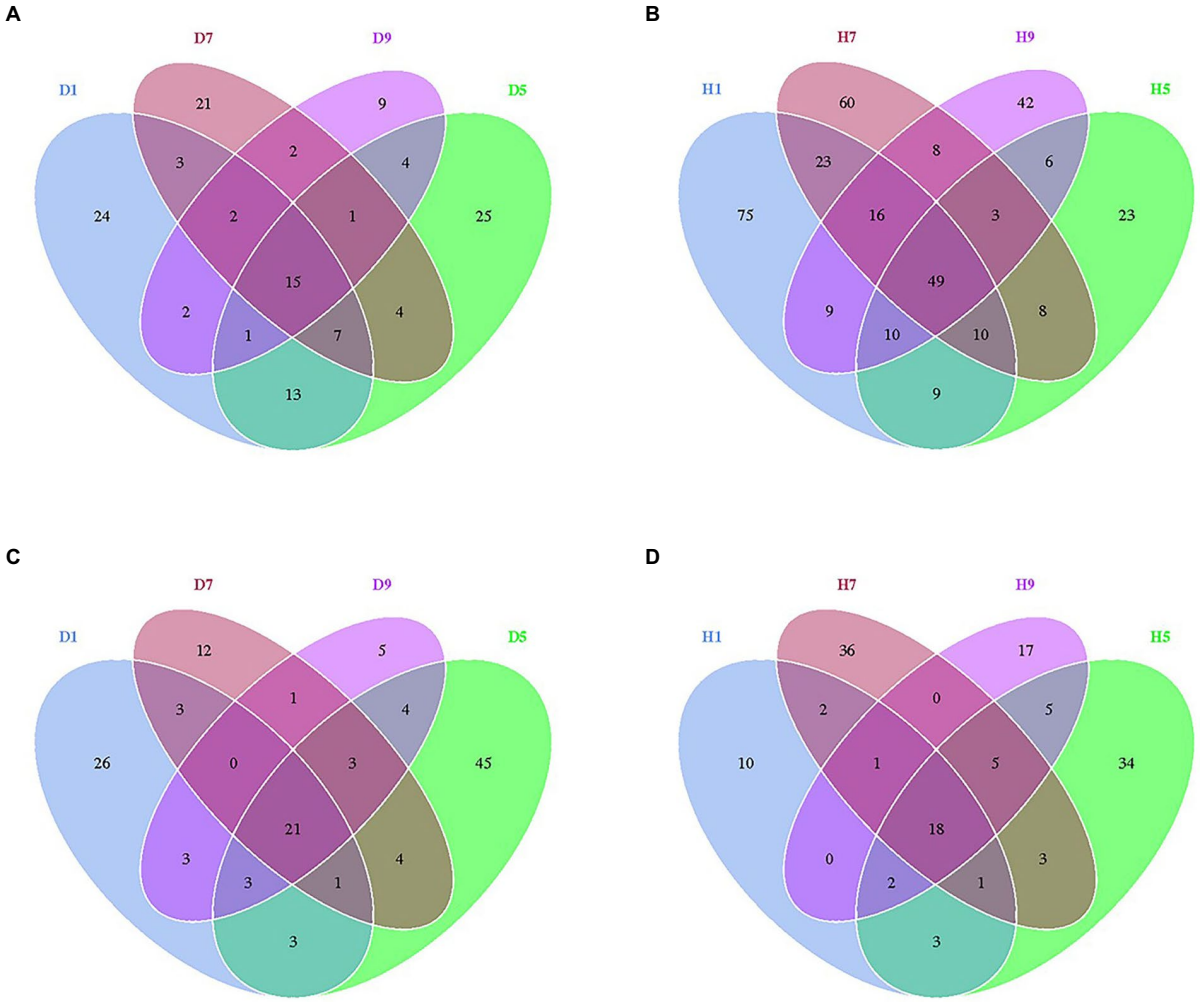
Venn diagram showing the number of fungal and bacterial OTUs observed in different groups at four different disease severity. Fungal Venn diagram in diseased **(A)** and healthy **(B)** groups at four different disease severities. Bacterial Venn diagram in diseased **(C)** and healthy **(D)** groups at four different disease severities. Numbers in the overlapping region indicated unique OTUs for two or three samples. Numbers in the non-overlapping regions indicated unique OTUs for the group.

At the OTU level, the distribution of OTUs among different disease severities was depicted with Venn diagrams (Figure 3). For fungal community, a total of 484 OTUs were obtained among which 133 and 351 belong to diseased and healthy samples, respectively (Figures 3A,B). The number of shared OTUs among the four diseased samples was 15. Only 24, 21, 9, and 25 OTUs were identified in D1, D5, D7 and D9 of the diseased sample, respectively. The number of shared OTUs among the four healthy samples was 49. Healthy samples H1, H5, H7, and H9 only included 75, 60, 42, and 23 unique OTUs, respectively. Notably, the number of fungal OTUs in diseased samples was lower than that in the healthy samples. The diversity index (shannon index) of diseased and healthy samples ranged from 0.46 ± 0.22 (mean ± SD) to 1.17 ± 0.88 and 2.86 ± 2.12 to 5.19 ± 0.43, respectively (Table 2). For diseased samples, the shannon index was the lowest and highest at a disease severity of 9 and 5, respectively. For healthy samples, the lowest and highest value was observed at S5 and S1, respectively. Noted that the shannon index of the four diseased samples and four healthy samples significantly differed. The richness index (chao 1 index) of diseased and healthy samples ranged from 23.19 ± 7.16 to 70.50 ± 10.50 and 101.46 ± 16.53 to 132.69 ± 45.76, respectively. For diseased samples, the chao 1 index was the lowest and highest at S9 and S5, respectively. For healthy samples, the chao 1 index was the lowest and highest at S5 and S1, respectively. The richness index (ACE index) of diseased and healthy samples ranged from 34.30 ± 7.56 to 74.42 ± 6.66 and 100.30 ± 16.38 to 144.71 ± 35.14, respectively. For diseased samples, the ACE index was the lowest and highest at S9 and S5, respectively. For healthy samples, the lowest and highest values were observed at S5 and S7, respectively. The shannon, chao 1 and ACE indices showed that the diversity and richness of the fungal community in healthy samples were higher than those in diseased samples (Table 2).

**Table 2:** Alpha-diversity indexes of fungal and bacterial community based on high-throughput sequencing in different samples.

	Disease severity groups	Diversity index	Richness index		Coverage index
		Shannon	Chao 1	ACE	goods_coverage
Fungi	D1	0.97 ± 0.72c	52.69 ± 24.18bcd	53.15 ± 18.27 cd	0.98 ± 0.01ab
	D5	1.17 ± 0.88c	70.50 ± 10.50abcd	74.42 ± 6.66bcd	0.97 ± 0.01ab
	D7	0.58 ± 0.24c	48.78 ± 23.35 cd	62.25 ± 32.70 cd	0.98 ± 0.01ab
	D9	0.46 ± 0.22c	23.19 ± 7.16d	34.30 ± 7.56d	0.99 ± 0.01a
	H1	5.19 ± 0.43a	132.69 ± 45.76a	143.95 ± 54.51a	0.94 ± 0.03c
	H5	2.86 ± 2.12b	101.46 ± 16.53abc	100.30 ± 16.38abc	0.96 ± 0.01bc
	H7	4.62 ± 0.33a	118.69 ± 16.51ab	144.71 ± 35.14a	0.95 ± 0.02c
	H9	3.93 ± 0.49ab	112.08 ± 21.03abc	115.44 ± 15.69ab	0.95 ± 0.01bc
Bacteria	D1	1.59 ± 0.29bc	61.75 ± 37.58a	72.35 ± 44.38a	0.99 ± 0.01a
	D5	3.06 ± 0.84a	67.14 ± 33.51a	64.92 ± 39.35a	0.98 ± 0.01a
	D7	2.55 ± 0.16ab	37.67 ± 12.41a	46.64 ± 17.54a	0.99 ± 0.01a
	D9	2.86 ± 0.40a	39.71 ± 19.63a	43.83 ± 22.12a	0.99 ± 0.01a
	H1	0.75 ± 0.60c	24.98 ± 8.42a	30.57 ± 12.04a	0.99 ± 0.01a
	H5	1.16 ± 1.18c	50.78 ± 54.06a	48.09 ± 44.75a	0.99 ± 0.01a
	H7	1.30 ± 0.58c	38.94 ± 24.26a	43.25 ± 26.77a	0.99 ± 0.01a
	H9	1.33 ± 0.58c	36.69 ± 15.64a	35.96 ± 14.33a	0.99 ± 0.01a

Data in column followed by the same letters were not significantly different at *p <*0.05 level according to Duncan’s new multiple range test.

For bacterial community, 271 OTUs were observed among which 134 and 137 belong to diseased and healthy samples, respectively (Figure 3). The four diseased and four healthy samples contained 21 and 18 shared OTUs, respectively. Diseased samples D1, D5, D7, and D9 contained 26, 12, 5, and 45 OTUs, respectively. Healthy samples H1, H5, H7, and H9 only include 10, 36, 17, and 34 unique OTUs, respectively. Notably, the number of bacterial OTUs in the diseased samples was lower than that in the healthy samples (Figures 3C,D). Similarly, the shannon index of diseased and healthy samples ranged from 1.59 ± 0.29 to 3.06 ± 0.84 and 0.75 ± 0.60 to 1.33 ± 0.58, respectively. The chao 1 index of diseased and healthy samples ranged from 37.67 ± 12.41 to 37.67 ± 12.41 and 24.98 ± 8.42 to 50.78 ± 54.06, respectively. The ACE index of diseased and healthy samples ranged from 43.83 ± 22.12 to 72.35 ± 44.38 and 30.57 ± 12.04 to 48.09 ± 44.75, respectively. Accordingly, for diseased samples, the lowest and highest values of the shannon, chao 1, ACE indices were observed at S1 and S5, S7 and S5, S9 and S1, respectively. For healthy samples, the lowest and highest values of the shannon, chao 1, ACE indices were observed at S1 and S9, S1 and S5, S1 and S5, respectively. In general, the Alpha-diversity index of the bacterial community in diseased samples was higher than that in healthy samples, except for groups D7 and D9.

### Fungal and bacterial community composition

#### Fungal community composition

**Figure 4:**
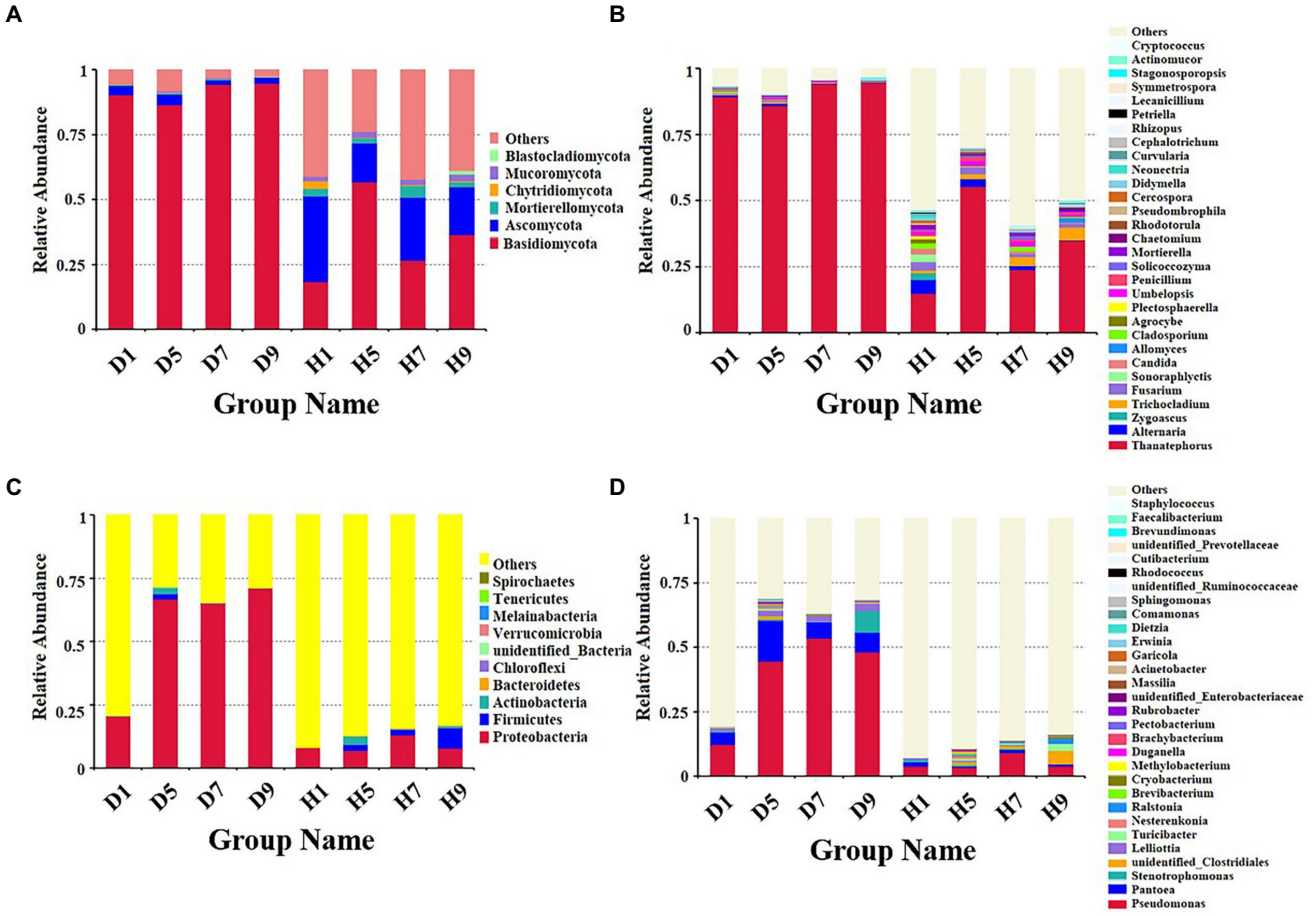
Microbial community composition of different groups at the phyla and genus levels. Fungal community composition of different groups at the phyla **(A)** and genus **(B)** levels. Bacterial community composition of different groups at the phyla **(C)** and genus **(D)** levels.

Based on the comparison of the sequencing and ITS data, all samples contain six phyla of fungi (Figure 4A): Basidiomycota, Ascomycota, Mortierellomycota, Chytridiomycota, Mucoromycota, and Blastocladiomycota. The dominant fungal phylum of diseased and healthy samples was Basidiomycota and Ascomycota, respectively, showing different relative abundance for four disease severities (Table 3). The relative abundance of Basidiomycota in the diseased groups (D1, D5, D7, D9) of four different disease severities were 90.43, 86.49, 94.48, and 94.72%, respectively. For the healthy groups (H1, H5, H7, H9), the relative abundance of Basidiomycota were 18.44, 56.76, 26.63, and 36.54%, respectively. The relative abundance of Ascomycota in diseased groups (D1, D5, D7, D9) of four different disease severities were 3.50, 4.04, 1.63, and 2.37%, respectively. For healthy groups (H1, H5, H7, H9), the relative abundances of Ascomycota were 32.84, 15.14, 24.16, and 18.39%, respectively. In summary, the results showed that the relative abundance of Basidiomycota in diseased samples was higher than that in healthy samples. In contrast, the relative abundance of Ascomycota in diseased samples was lower than that in healthy samples (Table 3).

**Table 3:** List of top 10 dominant taxa and their relative abundance in the fungal and bacterial community of the group.

Community Structure		Relative abundance (%)							
Fungi		D1	D5	D7	D9	H1	H5	H7	H9
Phylum	Basidiomycota	90.43 ± 7.67a	86.49 ± 10.69a	94.48 ± 2.66a	94.72 ± 2.79a	18.44 ± 8.03c	56.76 ± 39.97b	26.63 ± 11.34c	36.54 ± 18.21bc
	Ascomycota	3.50 ± 2.36 cd	4.04 ± 3.94 cd	1.63 ± 0.78d	2.37 ± 0.97d	32.84 ± 2.56a	15.14 ± 16.76bc	24.16 ± 7.69ab	18.39 ± 1.20b
	Mortierellomycota	0.49 ± 0.52b	0.54 ± 0.31b	0.39 ± 0.43b	0.15 ± 0.26b	2.96 ± 1.85ab	1.82 ± 1.97ab	4.54 ± 3.51a	1.97 ± 1.19ab
	Chytridiomycota	0.35 ± 0.37b	0.10 ± 0.09b	0.05 ± 0.09b	0.00 ± 0.00 b	3.06 ± 2.84a	0.10 ± 0.17b	0.49 ± 0.23b	0.25 ± 0.43b
	Mucoromycota	0.30 ± 0.51 c	0.59 ± 0.15abc	0.35 ± 0.34bc	0.15 ± 0.15c	1.63 ± 2.31abc	2.47 ± 1.41ab	2.07 ± 0.82abc	2.61 ± 2.01a
	Blastocladiomycota	0.00 ± 0.00a	0.00 ± 0.00a	0.00 ± 0.00a	0.10 ± 0.17a	0.00 ± 0.00a	0.00 ± 0.00a	0.00 ± 0.00a	1.58 ± 2.73a
	Others	4.93 ± 4.15d	8.23 ± 6.72 cd	3.11 ± 1.60d	2.51 ± 1.89d	41.07 ± 7.48ab	23.72 ± 20.55bc	42.11 ± 8.52a	38.66 ± 14.42ab
Genus	*Thanatephorus*	89.40 ± 8.49a	85.95 ± 1.12a	94.13 ± 2.53a	94.67 ± 2.81a	14.69 ± 7.91c	55.33 ± 41.93b	23.96 ± 10.38c	34.81 ± 18.48bc
	*Alternaria*	0.64 ± 0.43b	0.74 ± 0.78b	0.30 ± 0.30b	0.10 ± 0.17b	5.37 ± 3.47a	3.01 ± 4.31ab	1.28 ± 0.37b	0.30 ± 0.15b
	*Trichocladium*	0.30 ± 0.39c	0.74 ± 0.68c	0.25 ± 0.09c	0.25 ± 0.17c	1.13 ± 0.98c	1.92 ± 1.80bc	3.45 ± 1.76c	4.88 ± 1.29b
	*Fusarium*	0.44 ± 0.30bc	0.54 ± 0.56bc	0.20 ± 0.09c	0.00 ± 0.00c	3.16 ± 1.01a	2.47 ± 3.13ab	1.23 ± 0.56abc	1.38 ± 0.56abc
	*Cladosporium*	0.20 ± 0.09b	0.10 ± 0.17b	0.10 ± 0.09b	0.05 ± 0.09b	2.22 ± 0.92a	0.30 ± 0.26b	1.87 ± 2.12a	0.35 ± 0.23b
	*Sonoraphlyctis*	0.35 ± 0.37b	0.00 ± 0.00b	0.00 ± 0.00b	0.00 ± 0.00b	2.86 ± 2.81a	0.00 ± 0.00b	0.10 ± 0.09b	0.00 ± 0.00b
	*Candida*	0.10 ± 0.09b	0.10 ± 0.17b	0.05 ± 0.09b	0.00 ± 0.00b	2.12 ± 2.52a	0.10 ± 0.09b	0.69 ± 0.37ab	0.54 ± 0.48ab
	*Agrocybe*	0.49 ± 0.17ab	0.00 ± 0.00b	0.00 ± 0.00b	0.00 ± 0.00b	1.43 ± 2.23a	0.00 ± 0.00b	0.00 ± 0.00b	0.00 ± 0.00b
	*Zygoascus*	0.00 ± 0.00a	0.00 ± 0.00a	0.00 ± 0.00a	0.00 ± 0.00a	2.47 ± 4.14a	0.00 ± 0.00a	0.05 ± 0.09a	0.00 ± 0.00a
	*Allomyces*	0.00 ± 0.00a	0.00 ± 0.00a	0.00 ± 0.00a	0.10 ± 0.17a	0.00 ± 0.00a	0.00 ± 0.00a	0.00 ± 0.00a	1.58 ± 2.73a
	*Others*	8.09 ± 7.09d	11.83 ± 8.95 cd	4.98 ± 2.01d	4.83 ± 2.55d	64.55 ± 12.56a	36.88 ± 32.43bc	67.36 ± 13.81a	56.16 ± 17.56ab
**Bacteria**		**D1**	**D5**	**D7**	**D9**	**H1**	**H5**	**H7**	**H9**
Phylum	Proteobacteria	20.50 ± 2.15b	66.63 ± 24.72a	64.99 ± 18.49a	71.17 ± 16.20a	7.98 ± 7.91b	6.97 ± 5.25b	13.05 ± 8.52b	7.92 ± 2.70b
	Actinobacteria	0.03 ± 0.05a	2.53 ± 4.39a	0.10 ± 0.16a	0.03 ± 0.05a	0.00 ± 0.00a	3.39 ± 5.87a	0.29 ± 0.41a	0.98 ± 0.33a
	Firmicutes	0.06 ± 0.11b	2.15 ± 3.73b	0.16 ± 0.20b	0.00 ± 0.00b	0.03 ± 0.05b	2.41 ± 4.17b	2.19 ± 2.58b	7.89 ± 3.31a
	Bacteroidetes	0.06 ± 0.11a	0.13 ± 0.15a	0.06 ± 0.11a	0.00 ± 0.00a	0.00 ± 0.00a	0.16 ± 0.27a	0.29 ± 0.49a	0.06 ± 0.11a
	Chloroflexi	0.00 ± 0.00a	0.00 ± 0.00a	0.00 ± 0.00a	0.00 ± 0.00a	0.00 ± 0.00a	0.00 ± 0.00a	0.00 ± 0.00a	0.0006 ± 0.0011a
	Verrucomicrobia	0.00 ± 0.00a	0.00 ± 0.00a	0.0003 ± 0.0005a	0.00 ± 0.00a	0.00 ± 0.00a	0.00 ± 0.00a	0.00 ± 0.00a	0.00 ± 0.00a
	Melainabacteria	0.00 ± 0.00a	0.00 ± 0.00a	0.00 ± 0.00a	0.00 ± 0.00a	0.00 ± 0.00a	0.00 ± 0.00a	0.00 ± 0.00a	0.00 ± 0.00a
	Tenericutes	0.00 ± 0.00a	0.00 ± 0.00a	0.00 ± 0.00a	0.00 ± 0.00a	0.00 ± 0.00a	0.00 ± 0.00a	0.00 ± 0.00a	0.00 ± 0.00a
	Spirochaetes	0.00 ± 0.00a	0.00 ± 0.00a	0.00 ± 0.00a	0.00 ± 0.00a	0.00 ± 0.00a	0.00 ± 0.00a	0.00 ± 0.00a	0.00 ± 0.00a
	Others	79.34 ± 2.19a	28.52 ± 27.11a	34.63 ± 18.32b	28.80 ± 16.24b	91.98 ± 7.97a	87.04 ± 15.56a	84.19 ± 7.44a	83.08 ± 3.79a
Genus	*Pseudomonas*	12.23 ± 1.77b	44.52 ± 13.86a	53.61 ± 21.33a	48.23 ± 5.63a	3.74 ± 3.60b	3.30 ± 2.60b	9.16 ± 7.32b	3.83 ± 2.44b
	*Pantoea*	4.78 ± 2.95ab	15.91 ± 19.07a	6.08 ± 1.96ab	7.57 ± 4.42ab	1.84 ± 2.53b	0.54 ± 0.36b	1.17 ± 0.82b	0.73 ± 0.95b
	*Stenotrophomonas*	0.67 ± 1.07a	0.57 ± 0.53a	0.19 ± 0.16a	8.49 ± 13.72a	0.73 ± 0.65a	0.60 ± 0.54a	0.51 ± 0.45a	0.73 ± 0.95a
	*Lelliottia*	1.17 ± 0.88ab	2.15 ± 1.52ab	0.25 ± 0.31ab	2.76 ± 2.29a	0.51 ± 0.72ab	0.32 ± 0.40ab	0.41 ± 0.38ab	0.03 ± 0.05a
	*unidentified_Clostridiales*	0.00 ± 0.00b	1.20 ± 2.09b	0.00 ± 0.00b	0.00 ± 0.00b	0.00 ± 0.00b	1.30 ± 2.25b	1.05 ± 1.81b	0.51 ± 1.62a
	*Turicibacter*	0.00 ± 0.00b	0.67 ± 1.15b	0.00 ± 0.00b	0.00 ± 0.00b	0.00 ± 0.00b	0.86 ± 1.48b	0.63 ± 0.61b	2.79 ± 1.74a
	*Nesterenkonia*	0.00 ± 0.00a	0.86 ± 1.48a	0.00 ± 0.00a	0.00 ± 0.00a	0.00 ± 0.00a	1.17 ± 2.03a	0.00 ± 0.00a	0.00 ± 0.00a
	Ralstonia	0.00 ± 0.00b	0.25 ± 0.44b	0.00 ± 0.00b	0.00 ± 0.00b	0.06 ± 0.11b	0.29 ± 0.49b	0.44 ± 0.77b	2.12 ± 0.71a
	*Brevibacterium*	0.00 ± 0.00a	0.22 ± 0.38a	0.00 ± 0.00a	0.00 ± 0.00a	0.00 ± 0.00a	0.44 ± 0.77a	0.00 ± 0.00a	0.00 ± 0.00a
	*Cryobacterium*	0.00 ± 0.00b	0.22 ± 0.38ab	0.03 ± 0.05b	0.03 ± 0.05b	0.00 ± 0.00b	0.29 ± 0.49ab	0.25 ± 0.04ab	0.70 ± 0.38a
	*Others*	81.15 ± 1.66a	33.43 ± 25.35b	37.58 ± 19.35b	32.92 ± 14.56b	93.12 ± 7.42a	90.91 ± 11.19a	86.38 ± 5.53a	84.47 ± 4.31a

There was significant difference between different letters for the data in row direction (*p* < 0.05) according to Duncan’s new multiple range test.

**Figure 5:**
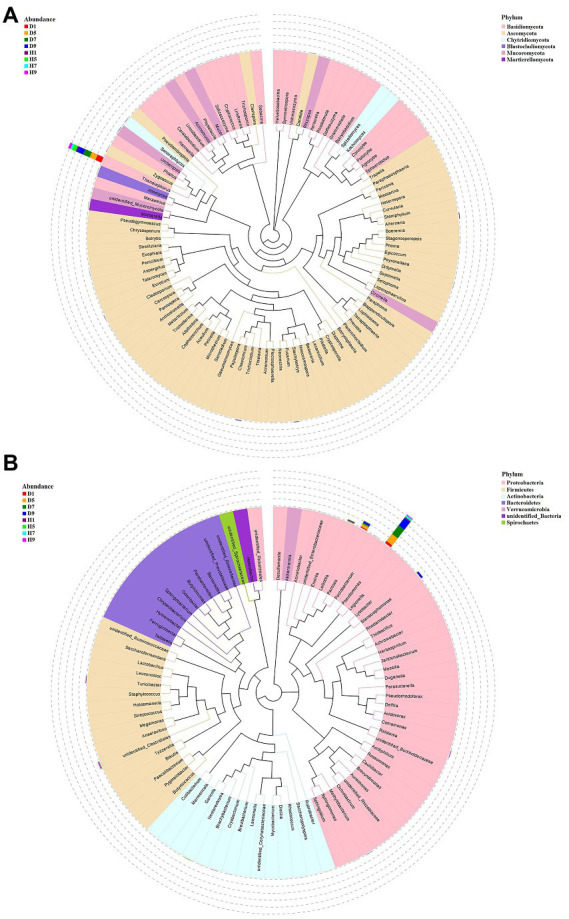
Maxium likelihood tree of the 100 most abundant fungal **(A)** and bacterial **(B)** genera in the eight group samples from tobacco target spot leaves. A color-coded bar plot shows the distribution of each fungal and bacterial genus in different groups.

The top 30 fungal genera at the genus level were displayed in Figure 4B. The top 10 genera were *Thanatephorus*, *Alternaria*, *Trichocladium*, *Fusarium*, *Cladosporium*, *Candida*, *Sonoraphlyctis*, *Zygoascus*, *Agrocybe*, and *Allomyces* (Table 3). The relative abundances of various genera in different leaf samples differ. The relative abundance of these genera in diseased samples were lower than those in healthy samples, except for *Thanatephorus* (the genera to which the pathogen of tobacco target spot belongs). *Thanatephorus* is the dominant genus in both diseased and healthy samples and the relative abundance of *Thanatephorus* in diseased samples and healthy samples significantly differ at different disease severities. The relative abundance of *Thanatephorus* showed a downward at S1 ~ S5 and then an upward trend at S5 ~ S9 in diseased samples and showed a peak at S9. However, in healthy samples, the relative abundance of *Thanatephorus* showed an upward at S1 ~ S5 and then a downward at S5 ~ S9 and showed a peak at S5. The relative abundance of *Thanatephorus* in diseased and healthy samples significantly differ at S5 compared to the other three disease severities samples. The top 100 genera were shown in the maximum-likelihood tree. The results showed that the dominant fungi belong to Basidiomycota, followed by Ascomycota. *Thanatephorus* was the dominant fungal genus of Basidiomycota. *Alternaria*, *Trichocladium*, and *Fusarium* were the dominant fungal genera of Ascomycota (Figure 5A).

#### Bacterial community composition

Based on the comparison of the sequencing and 16S datasets, all samples contain nine phyla of bacteria (Figure 4C): Proteobacteria, Actinobacteria, Firmicutes, Bacteroidetes, Chloroflexi, Verrucomicrobia, Melainabacteria, Tenericutes, and Spirochaetes. The dominant bacterial phyla of diseased and healthy leaf tissues are Proteobacteria, Actinobacteria, and Firmicutes, with different relative abundance associated with the four disease severities samples (Table 3). The relative abundance of Proteobacteria in the diseased groups (D1, D5, D7, and D9) at four different disease severities samples were 20.50, 66.63, 64.99, 71.17%, respectively. For healthy groups (H1, H5, H7, and H9), the relative abundance of Proteobacteria are 7.98, 6.97, 13.05, and 7.92%, respectively. The relative abundance of Actinobacteria in the diseased groups (D1, D5, D7, and D9) at four different disease severities samples were 0.03, 2.53, 0.10, and 0.03%, respectively. For healthy groups (H1, H5, H7, and H9), the relative abundance of Actinobacteria are 0.00, 3.39, 0.29, 0.98%, respectively. The relative abundance of Firmicutes in the diseased groups (D1, D5, D7, and D9) at four different disease severities samples were 0.06, 2.15, 0.16, 0.00%, respectively. For healthy groups (H1, H5, H7, and H9), the relative abundance of Firmicutes were 0.03, 2.41, 2.19, and 7.89%, respectively. In summary, the results showed that the relative abundance of Proteobacteria in diseased samples were higher than those in healthy samples and the relative abundance of Proteobacteria in diseased and healthy samples significantly differ at different disease severities (Table 3).

The top 30 bacterial genera at the genus level were displayed in Figure 4D and the top 10 genera at the genus level were displayed in Table 3: *Pseudomonas*, *Pantoea*, *Stenotrophomonas*, *Lelliottia*, *unidentified_Clostridiales*, *Turicibacter*, *Nesterenkonia*, *Ralstonia*, *Brevibacterium*, and *Cryobacterium*. Similarly, the relative abundance of various genera in different leaf samples differ. The relative abundance of these genera in diseased samples were lower than those in healthy samples, except for *Pseudomonas*, *Pantoea*, *Stenotrophomonas*, and *Lelliottia*. *Pseudomonas* and *Pantoea* were the dominant genera in both diseased and healthy samples. The relative abundance of *Pseudomonas* and *Pantoea* in diseased and healthy samples significantly differ at four disease severities. The top 100 genera were shown in the maximum-likelihood tree. The results showed that the dominant bacteria belong to Proteobacteria, followed by Actinobacteria and Firmicutes. *Pseudomonas* and *Pantoea* were the dominant bacterial genera of Proteobacteria. *Unidentified Clostridiales* and *Turicibacter* were the dominant bacterial genera of Firmicutes (Figure 5B).

### Spatial distribution of fungal and bacterial communities

**Figure 6:**
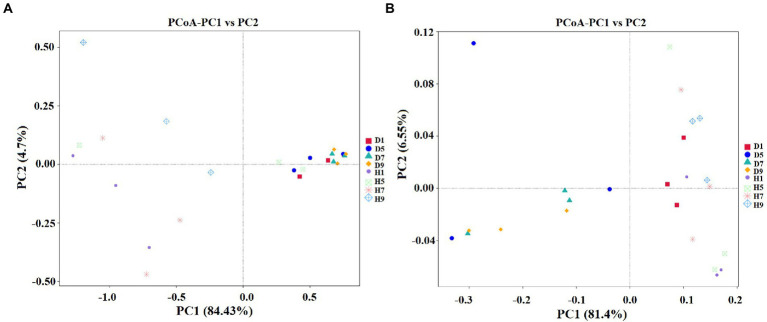
Principal Co-ordinate Analysis (PCoA) of the fungal **(A)** and bacterial **(B)** communities in the different group sample.

PCoA plots were used to reveal the spatial distribution of fungal and bacterial community (Figure 6). In fungial community, diseased groups (D1, D5, D7, and D9) with four disease severities were clustered together. In contrast, healthy groups (H1, H5, H7, and H9) with four disease severities were irregularly separated from each other (Figure 6A). For bacterial community, all diseased and healthy groups (D1, D5, D7, and D9; and H1, H5, H7, and H9) were irregularly separated from each other (Figure 6B). This indicated that there was a significant differences in the spatial distributions of fungal and bacterial communities in diseased and healthy samples.

### Significant differences in microbial communities

**Figure 7:**
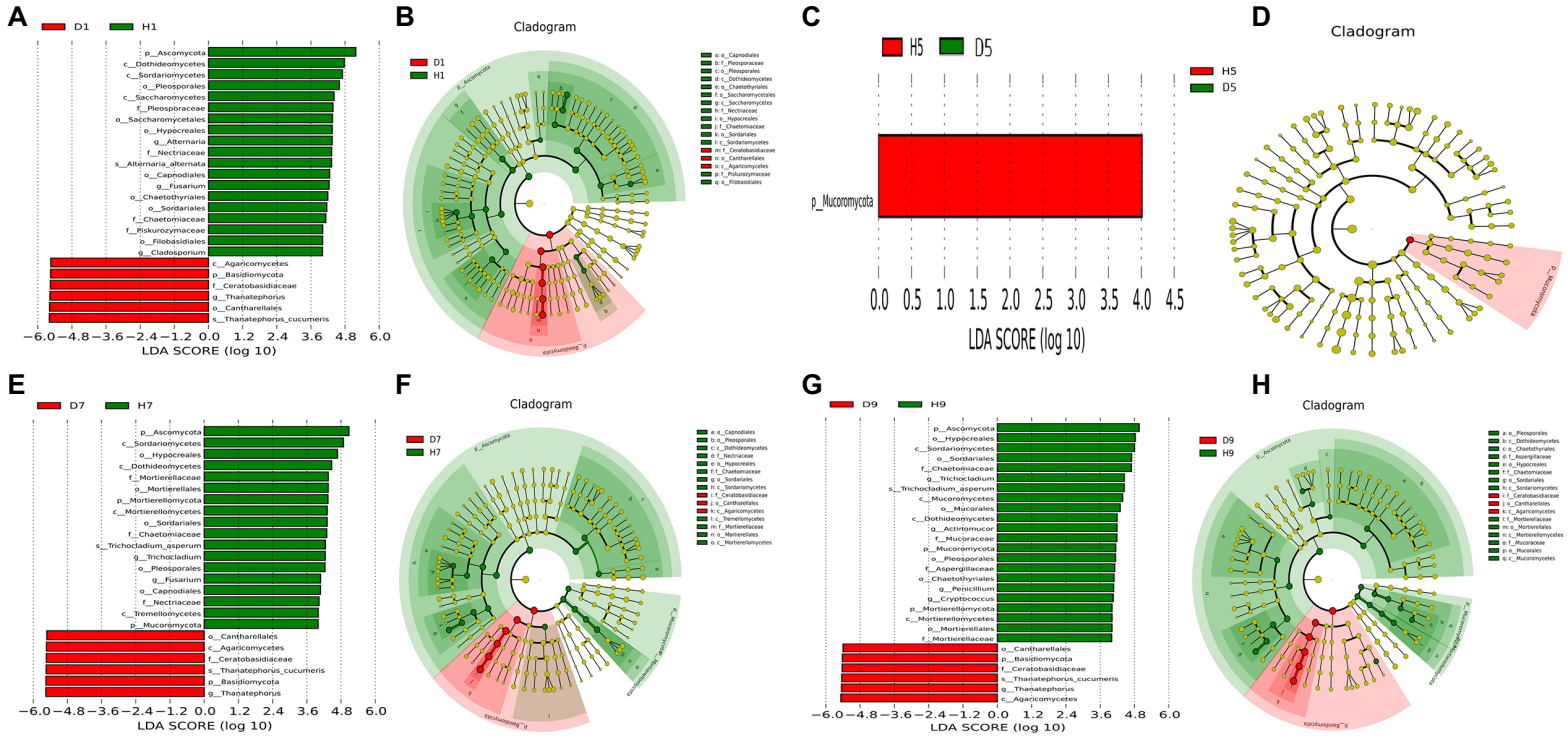
LEfSe analysis of fungi abundance between diseased and healthy samples of different disease severities. **(A)**, (**C**), **(E)**, (**G**) are LDA score identified the size of differentiation between diseased and healthy groups of 1, 5, 7, 9 disease severity, respectively, with a threshold value of 4. **(B)**, (**D**), **(F)**, (**H**) are the cladogram of microbial communities of 1, 5, 7, 9 disease severity, respectively.

**Figure 8:**
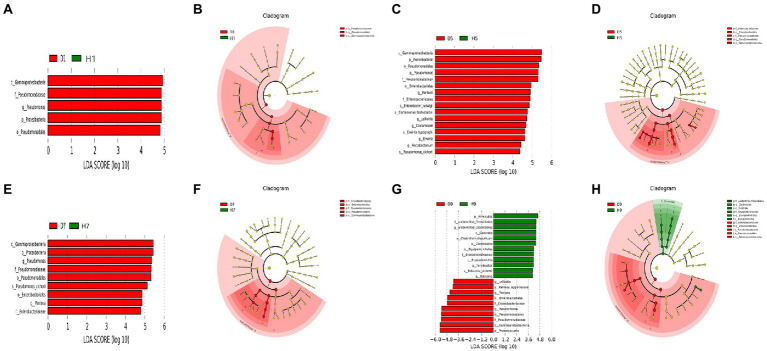
LEfSe analysis of bacteria abundance between diseased and healthy samples of different disease severities. **(A)**, (**C**), (**E**), **(G)** are LDA score identified the size of differentiation between diseased and healthy groups of S1, S5, S7, S9, respectively, with a threshold value of 4. **(B)**, **(D)**, **(F)**, **(H)** are the cladogram of microbial communities of S1, S5, S7, S9, respectively.

In order to determine the classified fungal and bacterial taxa with significant abundance differences between the diseased and healthy microbial communities of different disease severities, we performed biomarker analysis using the LEfSe method. In fungal community, 78 fungi clades present statistically significant differences with a LDA threshold of 4. In S1 leaf samples, 19 clades showed abundance advantage in healthy samples, while only 6 clades showed abundance advantage in diseased samples (Figures 7A,B). In S5 leaf samples, 1 clade showed abundance advantage in healthy samples, while 0 clade showed abundance advantage in diseased samples (Figures 7C,D). In S7 leaf samples, 18 clades showed abundance advantage in healthy samples, while 6 clades showed abundance advantage in diseased samples (Figures 7E,F). Most fungi were significantly enriched in S9 leaf samples, 22 clades showed abundance advantage in healthy samples, 6 clades showed abundance advantage in diseased samples (Figures 7G,H). Specifically, Asomycota (Phylum), Sordariomycetes (Class), Pleosoporales (Class), Hypocreales (Order), Sordariales (Order), Chaetomiaceae (Family) were enriched in 1, 7, 9 disease severities healthy samples. Agaricomycetes (Class), Basidiomycota (Phylum), Ceratobasidiaceae (Family), Cantharellales (Order), *Thanatephorus* (Genus), *Thanatephorus cucumeris* (Species) were enriched in 1, 7, 9 disease severities diseased samples. In bacterial community, 52 bacteria clades presented statistically significant differences with a LDA threshold of 4. In S1 leaf samples, 5 clades showed abundance advantage in diseased samples, while 0 clade showed abundance advantage in healthy samples (Figures 8A,B). In S5 leaf samples, 16 clades showed abundance advantage in diseased samples, while 0 clade showed abundance advantage in healthy samples (Figures 8C,D). In S7 leaf samples, 9 clades showed abundance advantage in diseased samples, while 0 clade showed abundance advantage in healthy samples (Figures 8E,F). In S9 leaf samples, 10 clades showed abundance advantage in diseased samples, 12 clades showed abundance advantage in healthy samples (Figures 8G,H). Specifically, Gammaproteobacteria (Class), Pseudomonadaceae (Family), *Pseudomonas* (Genus), Proteobacteria (Phylum), Pseudomonadales (Order) were enriched in S1, S5, S7, S9 diseased samples. Firmicutes (Phylum), *Clostridia* (Genus), *Clostridium disporicum* (Species), Clostridiales (Order), Erysipelotrichales (Order), Erysipelotrichaceae (Family), Erysipelotrichia (Class), *Turicibacter* (Genus), *Ralstonia pickettii* (Species), *Ralstonia* (Genus) were enriched in S9 healthy samples.

### Fungal and bacterial functional characteristics

**Figure 9:**
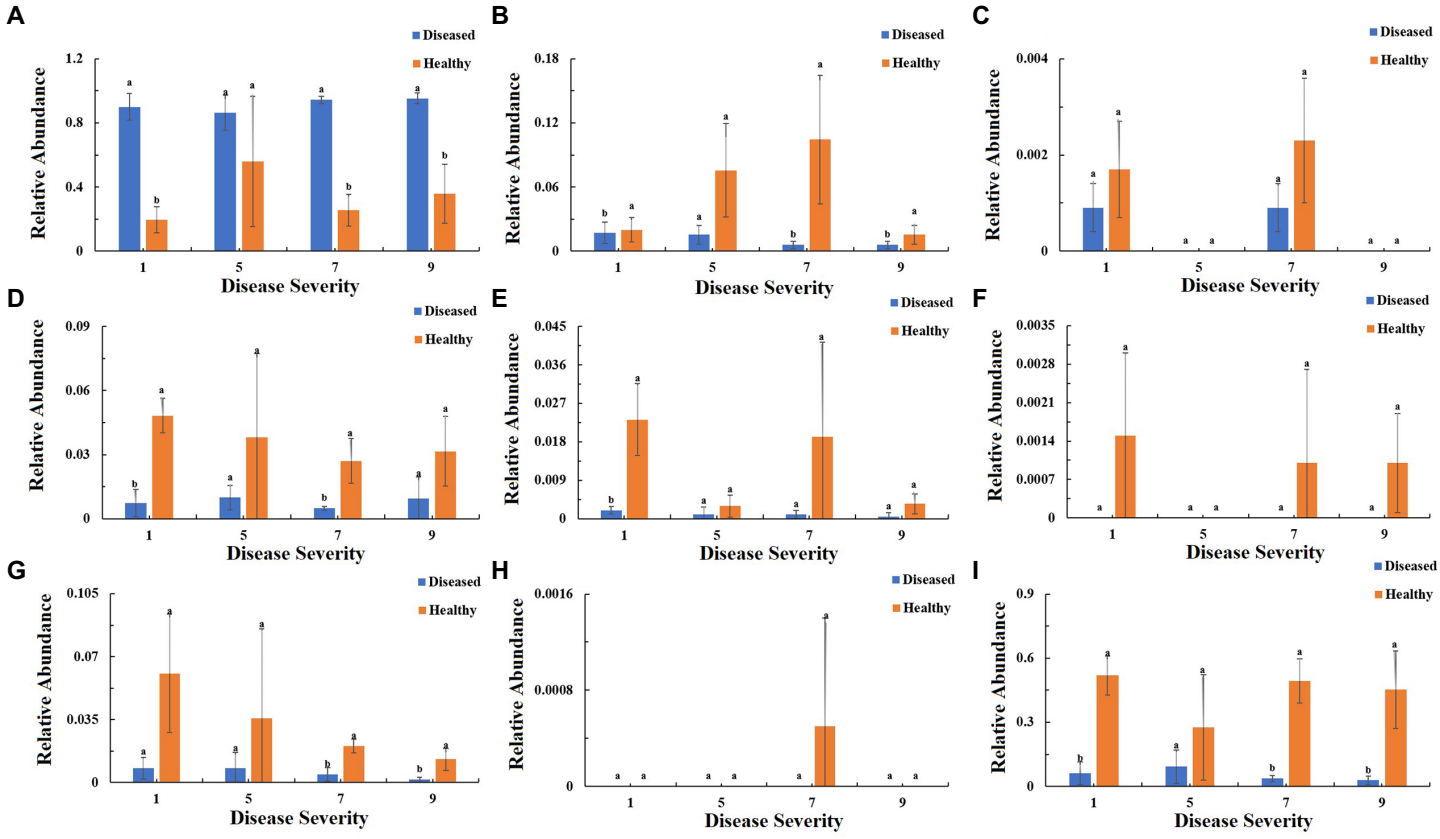
Relative abundance of fungal functional groups (modes) based on OTU annotation table with disturbance frequency level. **(A)** Pathotroph, **(B)** Saprotroph, **(C)** Symbiotroph, **(D)** Pathotroph-Saprotroph, **(E)** Pathotroph-Symbiotroph, **(F)** Pathogen-Saprotroph-Symbiotroph, **(G)** Pathotroph-Saprotroph-Symbiotroph, **(H)** Saprotroph-Symbiotroph, **(I)** Unassigned.

To understand the function of the fungal community in tobacco leaves, the trophic modes of fungi of tobacco leaves with four disease severities were predicted using the FUNGuid database (Figure 9). The relative abundance of pathotroph mode in healthy samples was lower than that in diseased samples, and there were significant difference between diseased and healthy samples at S1, S7, S9 (Figure 9A). The relative abundance of saprotroph mode showed little change in diseased samples with the increase of disease severity, and there were significant difference between diseased and healthy samples at S1, S7, and S9. In healthy samples, there was a peak at S7 (Figure 9B). The relative abundance of symbiotroph mode was detectable in diseased and healthy leaf tissues at S1 and S7, but not detectable at S5 and S9 (Figure 9C). Pathotroph–saprotroph mode abundance in diseased and healthy samples was relatively constant with the increase of disease severity, and there were significant difference at S1 and S7 (Figure 9D). Pathotroph–symbiotroph mode abundance in diseased samples was lower than that in healthy samples, and there was a significant difference between diseased and healthy samples at S1, there were peaks at S1, S7 in healthy samples (Figure 9E). Pathogen–saprotroph–symbiotroph mode abundance was detectable in healthy samples at S1, S7 and S9, but not detectable in diseased samples at any disease severity leaf samples (Figure 9F). The relative abundance of pathoproth–saprotroph–symbiotroph mode gradually decreased from S1 to S9 in healthy samples and there were significant difference between diseased and healthy samples at S7 and S9 (Figure 9G). Saprotroph–symbiotroph mode abundance was only detectable in healthy samples at S7 (Figure 9H). The relative abundace of unassigned mode in diseased samples was lower than that in healthy samples and there were significant difference between diseased and healthy samples at S1, S7 and S9 (Figure 9I).

**Figure 10:**
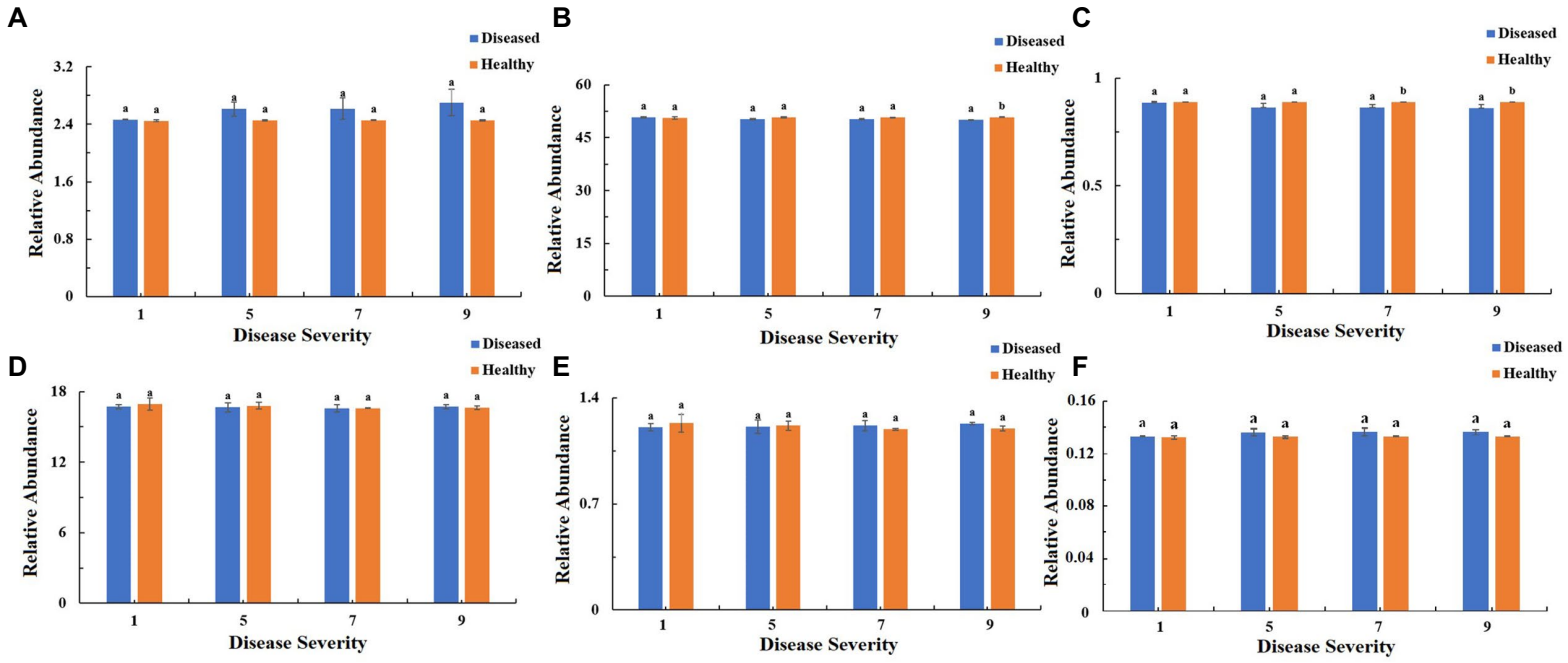
PICRUSt analyses of the changes in the KEGG level 1 functional categories of bacteria in tobacco leaf samples. **(A)** Cellular Processes, **(B)** Metabolism, **(C)** Organismal Systems, **(D)** Genetic Information Processing, **(E)** Human Diseases, **(F)** Environmental Information Processing.

**Figure 11:**
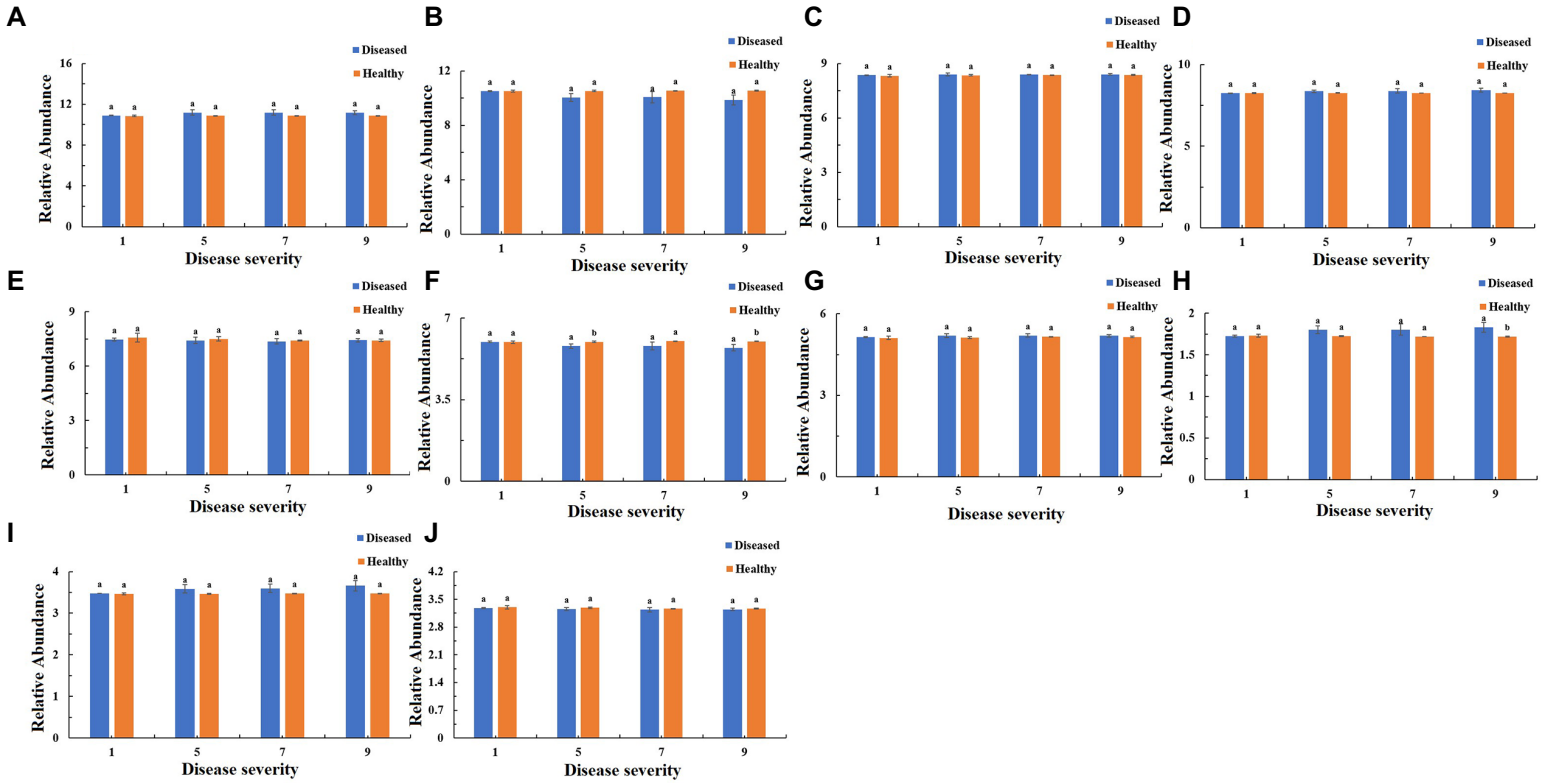
PICRUSt analyses of the changes in the KEGG level 2 functional categories of bacteria in tobacco leaf samples. **(A)** Membrane Transport, **(B)** Energy Metabolism, **(C)** Carbohydrate Metabolism, **(D)** Amino Acid Metabolism, **(E)** Replication and Repair, **(F)** Metabolism of Cofactors and Vitamins, **(G)** Poorly Characterized, **(H)** Translation, **(I)** Cellular Processes and Signaling, **(J)** Nucleotide Metabolism.

**Figure 12:**
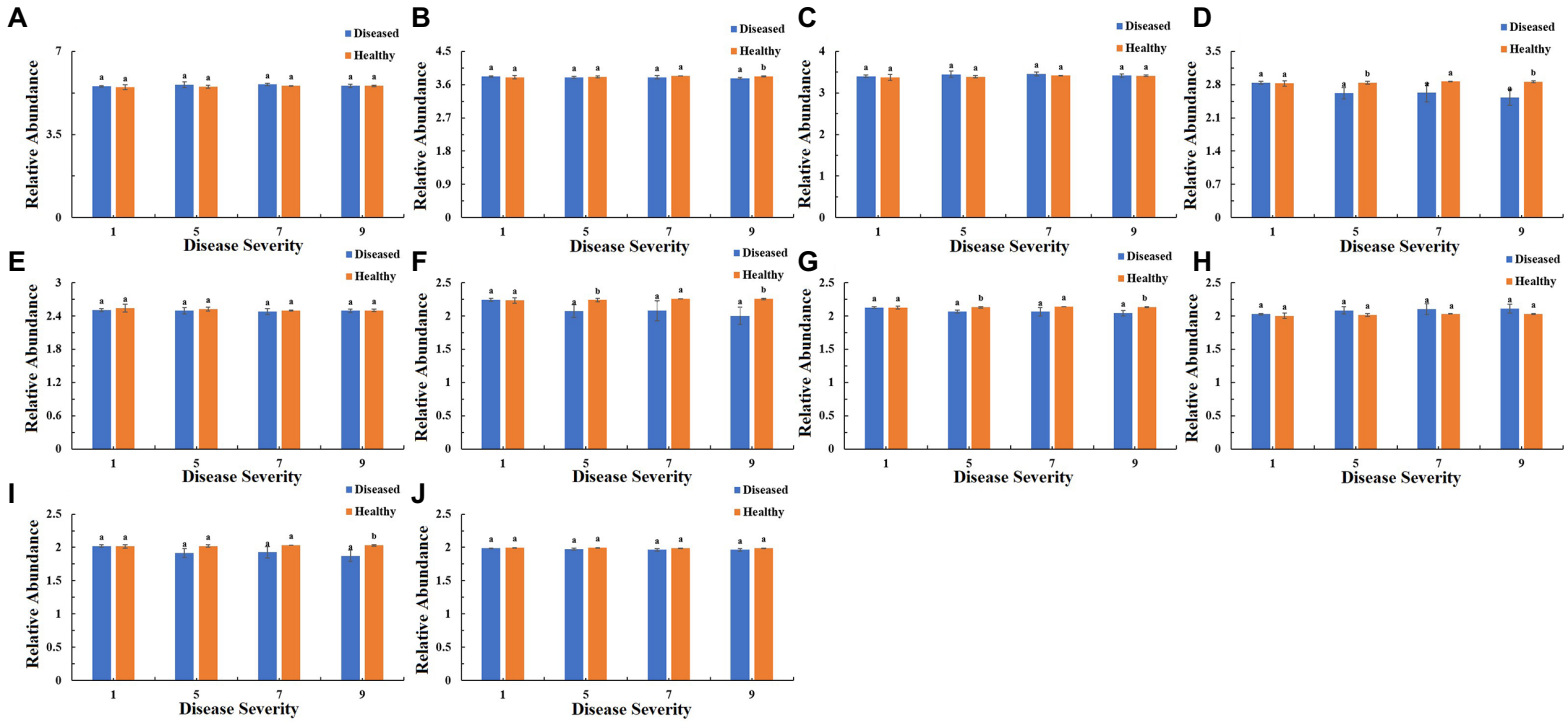
PICRUSt analyses of the changes in the KEGG level 3 functional categories of bacteria in tobacco leaf samples. **(A)** Transporters Ribosome, **(B)** General function prediction only, **(C)** ABC transporters, **(D)** Photosynthesis proteins, **(E)** DNA repair and recombination proteins, **(F)** Photosynthesis, **(G)** Peptidases, **(H)** Two component system, **(I)** Porphyrin and chlorophyll metabolism, **(J)** Purine metabolism.

The function of the bacteria community of tobacco leaves was predicted for four disease severities by utilizing the PICRUSt database (Figures 10–12). The relative abundance of the level 1 categories of the KEGG pathways for diseased and healthy samples fluctuated with the increase of disease severity, but with no clear patterns, and there were significant difference between diseased and healthy samples in metabolism at S9, and in organismal systems at S7 and S9 (Figure 10). This was similar for level 2 categories, and there were significant difference between diseased and healthy samples in metabolism of cofactors and vitamins at S5 and S9, and in translation at S9 (Figure 11). Similar results were observed for level 3 categories, and there were significant difference between diseased and healthy samples in general function prediction only at S9, and in photosynthesis proteins at S5 and S9, and in photosynthesis at S5 and S9, and in peptidases at S5 and S9, and in porphyrin and chlorophyll metabolism at S9 (Figure 12). In summary, most bacterial gene sequences seem to be independent of the disease severity.

### Fungal and bacterial co-occurrence

**Figure 13:**
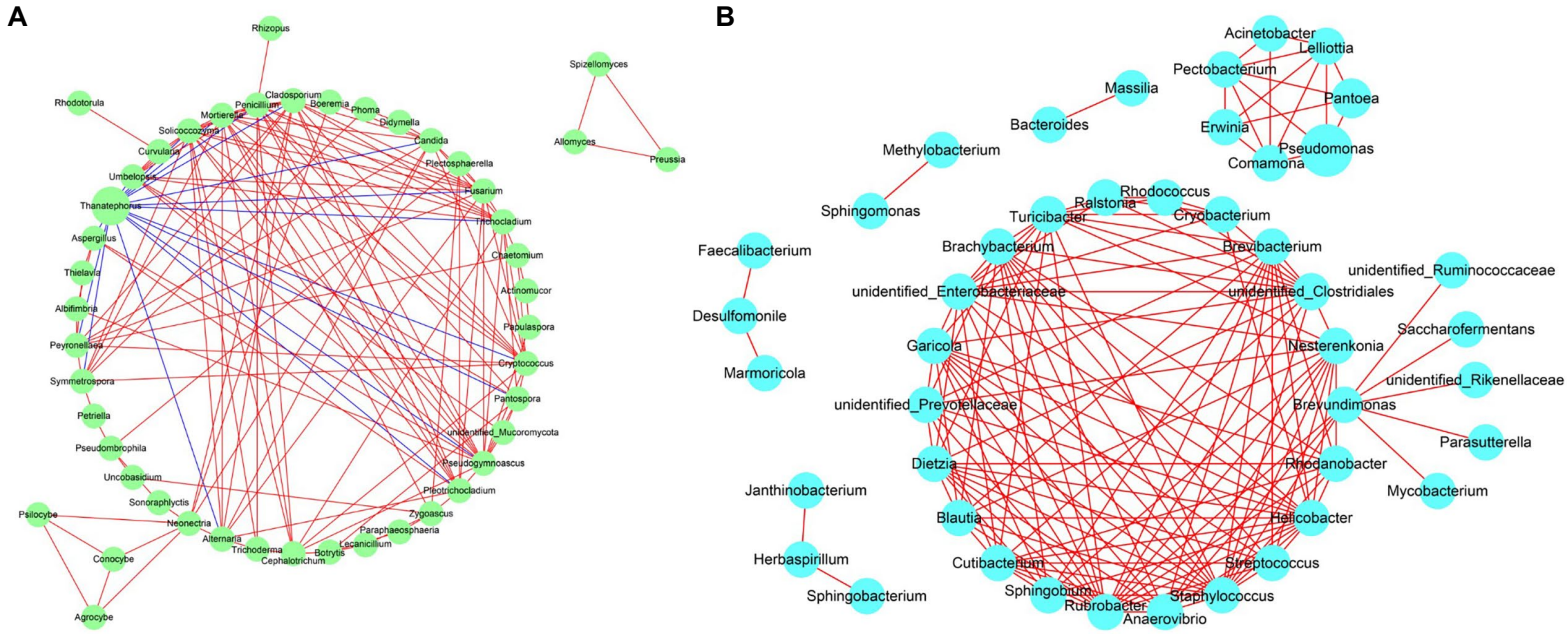
Network analysis of fungal **(A)** and bacterial **(B)** taxa showing significant positive or negative co-occurrences based on Spearman’s rank correlations. Red lines represent positive correlations and blue lines represent negative correlations.

Network analysis of the co-occurrence of the most abundant 50 fungial species revealed that there were 49 fungal species showing positive co-occurrence with each other, except for *Thanatephorous* showed a highly significant negative co-occurrence with 16 genus including *Fusarium*, *Pantospora*, *Alternaria*, *Symmetrospora* etc. (*p* < 0.05, *r* < −0.5) (Figure 13A). Network analysis of the co-occurrence of the most abundant 50 bacterial species revealed that all 50 bacterial species showed positive co-occurrence with at least one other bacterial species (*p* < 0.05, *r* > 0.5) (Figure 13B). There was no bacterial species to show negative co-occurrence with at least one other bacterial species.

## Discussion

Microbes play an important role in plant growth and development and resistance to biotic and abiotic stresses (Luo et al., 2017). Simultaneously, phyllosphere microbes play important roles in maintaining leaf health and ecosystem balance (Baker et al., 2010). In this study, the V3–V4 hypervariable regions of the 16S rRNA and ITS region of rDNA were amplified to determine the effect of the disease severity of tobacco target spot on microbial diversity. The results of a previous study showed that disease severity reduced the diversity of microbial communities (Manching et al., 2014). The results of this study also showed that the shannon, chao 1, ACE indices decreased with the increase of disease severity. Similarly, based on a study on bacterial diversity in needles with shoot blight with different disease severities, the diversity and abundance of endophytic bacteria gradually decreased with the aggravation of the disease (Xie et al., 2021). Shen et al. reported that different tissues of eucalyptus were differently affected by diseases, which was consistent with the conclusion drawn in this study about the Alpha-diversity of healthy tissues was higher than that of diseased tissues (Shen et al., 2016).

Pathogen stimulation has a significant effect on the composition of the microbial community (Erlacher et al., 2014). The ITS dataset showed that Basidiomycota was the dominant phylum in both diseased and healthy samples at all four disease severities. The relative abundance of Basidiomycota gradually increased with the increase of disease severity, whereas the relative abundance of Ascomycota gradually decreased with the increase of disease severity, similar to the trend reported by Zhang et al. (2018) when studying pumpkin leaves affected by powdery mildew. The dominant fungal genus of both diseased and healthy leaves was *Thanatephorous*, which was consistent with the research results obtained for culturable fungi isolation by tissue isolation (Hou et al., 2018). The relative abundance of *Thanatephorous* positively correlated with the disease severity in both diseased and healthy samples, which is consistent with the results reported by Zhang et al. (2018) who pointed out that the abundance of *Podosphaere* (pathogen of pumpkin powdery mildew) increased with the increase of disease severity. The results of this study showed that the relative abundance of eight genera in diseased samples decreased from S1 to S9, except for *Thanatephorous* and *Allomyces*. For healthy samples, the relative abundance of six genera increased from S1 to S9, except for *Thanatephorous*, *Trichocladium*, *Agrocybe*, and *Zygoascus*. The above-mentioned two phenomena indicated the existence of synergistic or antagonistic relationships between *Thanatephorous* and the other nine genera. This has also been pointed out in previous studies. For example, Arnold et al. reported that foliar fungi can alter the plant disease severity (Arnold et al., 2003). Simultaneously, Busby et al. pointed out that *Cladosporium*, *Trichoderma*, and *Epicoccum* can alter the disease severity caused by the *Melampsora rust* (Busby et al., 2016).

The 16S dataset showed that Proteobacteria represent the dominant phylum in both diseased and healthy samples at all disease severities. The relative abundance of Proteobacteria gradually increased with the increase of disease severity in diseased samples, whereas it decreased with the increase of disease severity in healthy samples, which was similar to the trend reported by Xie et al. (2021) when studying the bacterial diversity in needles with different disease severities of shoot blight. The dominant bacterial genus of both diseased and healthy leaves was *Pseudomonas*, followed by *Pantoea*, which was in agreement with the results of previous studies of phyllospheric microorganisms of tobacco (Liu et al., 2020; Chen et al., 2021). It is of significance that the relative abundance of *Pseudomonas* and *Pantoea* correlate with the disease severity. Their relative abundance increased with the disease severity increasing. One can speculate that they may have a coevolutionary effect with pathogenic *Thanatephorous*. Genus *Pseudomonas* can cause diseases in tobacco; for example, *Pseudomonas syringae* pv*. tabaci* has been reported to cause tobacco wildfire (Wolf and Foster, 1918) and *Pseudomonas syringae* pv*. angula* causes tobacco angular leaf spot (Cole, 1960). Simultaneously, *Pantoea agglomerans* has also been reported to cause bacterial soft rot in Chinese cabbage (Guo et al., 2019). In this study, several potential bacterial pathogens, such as biocontrol bacterium *Paenibacillus*, were identified by high-sequence analysis (Tsai et al., 2022). *Paenibacillus polymyxa* reportedly protects *Arabidopsis thaliana* against pathogens and abiotic stress (Salme et al., 2005). Based on the prediction of the bacterial function, the cell motility, amino acid metabolism, lipid metabolism, cellular processes, and signaling in diseased samples increased with the disease severity increasing, which may be caused by the invasion of pathogenic *T. cucumeris* (Luo et al., 2017).

It is worth noting that the turning point of the relative abundance change of *Thanatephorous* at S5 and the LEfSe analysis showed that *T. cucumeris* was the most abundant biomarker in diseased samples at S1, S7 and S9. It is speculated that the pathogen propagates in large numbers after colonization, causing leaf spots on the tobacco leaves. Other phyllosphere microorganisms compete with the pathogen for ecological niches at S5. There are two situations. One is that the pathogen occupies more ecological niches (the relative abundance increases), which increases the disease severity of tobacco leaves. The second is that pathogen occupies less ecological niches (the relative abundance decreases), and the disease severity of tobacco leaves remains unchanged (Wintermute and Silver, 2010; Perazzolli et al., 2014). In this study, *Thanatephorus* showed no positive co-occurrence with any fungal genera but did have a negative co-occurrence with *Fusarium, Pantospora, Alternaria,* etc., which may because *Thanatephorus* greatly perturbed the resident fungal microbiota (Hassani et al., 2018). In bacterial community, there was no bacterial species shown any negative co-occurrence with each other, which meant that the most bacterial did not exert major effects on each other.

## Conclusion

The diversity and abundance of microorganisms in tobacco leaves were strongly affected by the tobacco target spot disease severity. The structure of fungal and bacterial community were consistent with the changes caused by tobacco target spot pathogen. A genus with high relative abundance in the fungal community was *Thanatephorus* and genera with high relative abundance in the bacterial community were *Pseudomonas* and *Pantoea*. The infestation with the tobacco target spot fungus affected the community structure and diversity of phyllosphere bacteria and fungi in tobacco. The fungal diversity and relative abundance increased with the tobacco target spot disease severity increasing. The bacterial diversity index decreased with the tobacco target spot disease severity increasing.

## Data availability statement

The datasets presented in this study can be found in online repositories. The names of the repository/repositories and accession number(s) can be found at: https://www.ncbi.nlm.nih.gov/, PRJNA887197 https://www.ncbi.nlm.nih.gov/, PRJNA887218.

## Author contributions

MS, CS, HW, and FW contributed to conception and design of the study. MS, HW, FL, JL, LC, and LX conducted the experiment and collected the samples. MS performed the analysis of samples. MS analyzed the data. MS wrote the first draft of the manuscript. CS and HW wrote sections of the manuscript. MS, HW, CS, YH, and FW revised the Error free manuscript. All authors contributed to the article and approved the submitted version.

## Funding

This work was supported by Guizhou Science Technology Foundation [ZK(2021)Key036], the National Natural Science Foundation of China (32160522, 31960550), China National Tobacco Corporation (110202001035(LS-04), 110202101048(LS-08), 110202101045(LS-05)), ‘Hundred’ Level Innovative Talent Foundation of Guizhou Province [GCC(2022)028-1], Guizhou Provincial Academician Workstation of Microbiology and Health [(2020)4004], International Science and Technology Cooperation Base [(2020)4102], and Guizhou Tobacco Company (2020XM22, 2020XM03). The authors declare that this study received funding from the China National Tobacco Corporation and Guizhou Tobacco Company. The funders were not involved in the study design, collection, analysis, interpretation of data, the writing of this article or the decision to submit it for publication.

## Conflict of interest

The authors declare that the research was conducted in the absence of any commercial or financial relationships that could be construed as a potential conflict of interest.

## Publisher’s note

All claims expressed in this article are solely those of the authors and do not necessarily represent those of their affiliated organizations, or those of the publisher, the editors and the reviewers. Any product that may be evaluated in this article, or claim that may be made by its manufacturer, is not guaranteed or endorsed by the publisher.

## Footnotes

^1^ http://huttenhower.sph.harvard.edu/lefse/

^2^ https://cytoscape.org/
